# Anatomical and functional plasticity in early blind individuals and the mixture of experts architecture

**DOI:** 10.3389/fnhum.2014.00971

**Published:** 2014-12-17

**Authors:** Andrew S. Bock, Ione Fine

**Affiliations:** Department of Psychology, University of WashingtonSeattle, WA, USA

**Keywords:** blind, anophthalmia, functional connectivity, anatomical connectivity, fMRI, DTI, plasticity, review

## Abstract

As described elsewhere in this special issue, recent advances in neuroimaging over the last decade have led to a rapid expansion in our knowledge of anatomical and functional correlations within the normal and abnormal human brain. Here, we review how early blindness has been used as a model system for examining the role of visual experience in the development of anatomical connections and functional responses. We discuss how lack of power in group comparisons may provide a potential explanation for why extensive anatomical changes in cortico-cortical connectivity are not observed. Finally we suggest a framework—cortical specialization via hierarchical mixtures of experts—which offers some promise in reconciling a wide range of functional and anatomical data.

## Introduction

“Now there was among the Thebans a soothsayer, Tiresias, son of Everes and a nymph Chariclo, of the family of Udaeus, the Spartan, and he had lost the sight of his eyes … Pherecydes says that he was blinded by Athena; for Chariclo was dear to Athena… and Tiresias saw the goddess stark naked, and she covered his eyes with her hands, and so rendered him sightless. And when Chariclo asked her to restore his sight, she could not do so, but by cleansing his ears she caused him to understand every note of birds; and she gave him a staff of cornel-wood, wherewith he walked like those who see.” (Apollodorus, C2nd A.D.)

Since the 1960”s blindness due to peripheral causes has provided a classic model system for understanding prenatal, postnatal and adult cortical plasticity in animals (e.g., Wiesel and Hubel, 1965a,b). Some of the appealing properties of early blindness as a model is that the confounding effects of cortical pathology are minimized, the connectivity and functional properties of the human and primate visual system are relatively well-understood compared to other cortical areas, and at least a fifth of the brain is normally devoted to visual processing, thereby offering the opportunity to study large-scale changes in functional and anatomical connectivity. This review focuses on modifications in structural connectivity and functional responses resulting from the early loss of vision in humans, with the goal of linking these data to changes in neural selectivity.

One of the most interesting aspects of blindness is that both deterioration and enhancement are observed. With respect to deterioration, early blindness results in dramatic changes in the neuroanatomy of subcortical and cortical visual areas. Early blindness results in attenuation of the connections between the eye and early visual cortex (discussed below), substantial reductions in the size of the LGN and V1 (Wiesel and Hubel, 1963; for review see Movshon and Van Sluyters, 1981; Dehay et al., 1989, 1996a; Bridge et al., 2009; Karlen and Krubitzer, 2009), alterations within the neuronal structure (Heumann and Rabinowicz, 1982; Olavarria et al., 2008; Gabbott and Stewart, 2012), and neurochemistry of early visual cortex (Fosse et al., 1989; Benevento et al., 1995; Dehay et al., 1996b; Desai et al., 2002; Morales et al., 2002; Weaver et al., 2013). Functionally, there is a loss of ability to perceive and understand the visual world if vision is ever restored (Fine et al., 2003; Ostrovsky et al., 2006, 2009) and impaired performance for certain auditory and tactile spatial tasks (e.g., Sterr et al., 2003; Gori et al., 2014).

Concerning enhancement, a wide range of studies suggest that blind individuals show superior performance on and/or occipital BOLD responses to a variety of relatively low level auditory (Lessard et al., 1998; Röder et al., 1999; Gougoux et al., 2004, 2005; Voss et al., 2004) and tactile (Van Boven et al., 2000; Goldreich and Kanics, 2003; Alary et al., 2009) tasks. In addition, early blind subjects show enhanced performance on and occipital BOLD responses for a variety of higher levels tasks involving attention (Kujala et al., 2000; Weaver and Stevens, 2007) memory (Röder et al., 2001; Amedi et al., 2003; Raz et al., 2005, 2007; Burton et al., 2010), and executive control (Bedny et al., 2013).^1^

Here we focus on alterations in anatomical connectivity and functional correlations due to early blindness, while relating these findings to the wider literature of how blindness affects functional responses. One source of puzzlement in recent years has been that although early blindness clearly results in an *enhancement* of auditory and tactile responses within occipital cortex, it appears to *reduce* functional correlations between occipital and auditory/somatosensory areas. Here we suggest a framework—cortical specialization via hierarchical mixtures of experts—which offers some promise in being able to reconcile this apparent contradiction. Briefly, the mixture of experts (ME) modular architecture consists of a gating network that mediates the competition between a number of “experts” (or modules) to learn different tasks (Jacobs et al., 1991). The expert network performs two roles: gating the output of each expert network such that the most accurate expert has a larger influence on the final response than less accurate experts, and modulating learning, so as to guide the expert networks towards dividing the task-space. The ME architecture can potentially explain a surprisingly wide set of findings on the effects of blindness. As far as task-related BOLD responses are concerned, the ME architecture can explain why a wide variety of tasks elicit cross-modal responses, yet responses seem to be extremely task-specific, how cross-modal plasticity often takes advantage of underlying computational specializations (e.g., motion processing in hMT+, face processing in fusiform cortex), and why losses of functionality in non-deprived areas are occasionally observed. The ME architecture also explains why there are lowered resting state functional correlations and indications of reduced anatomical connectivity between occipital and non-deprived sensory areas. Finally the ME architecture can explain findings of enhanced resting state functional correlations between occipital and pre-frontal and frontal areas and increased white matter volume in the tracts connecting occipital and pre-frontal areas.

## Anatomical connectivity

We assume the reader has a working knowledge of assessing anatomical connectivity using diffusion weighted imaging, for a review we recommend Mori and Zhang (2006) and Jones et al. (2013). Briefly, diffusion weighted imaging allows for the non-invasive mapping of the diffusion of water molecules. Because water diffuses more freely along than across axonal fiber membranes, these diffusion patterns can be used to indirectly measure anatomical connectivity on a gross scale. These diffusion patterns are often approximated as an ellipse, with longitudinal diffusivity representing diffusion along the principal (most elongated) axis, and radial diffusivity representing the mean diffusion along the two minor axes. The ellipse can also be described in terms of fractional anisotropy, which combines longitudinal and radial measures into a single value where 0 represents a sphere and values approaching 1 represent an elongated ellipse. Generally, higher longitudinal and/or fractional anisotropy values are often interpreted as reflecting potential increases in anatomical connectivity. Conversely, lower fractional anisotropy and longitudinal diffusivity values and/or increases in radial diffusivity can be due to decreases in anatomical connectivity. However, diffusion data (in the absence of other measures, see Stikov et al., 2011) cannot differentiate the effects of a number of factors, including fiber diameter, fiber density, membrane permeability, myelination and the presence of crossing fibers (or more generally intra-voxel orientation coherence Bock et al., 2010, see Jones et al., 2013 for a review).

### Subcortical

**Figure 1:**
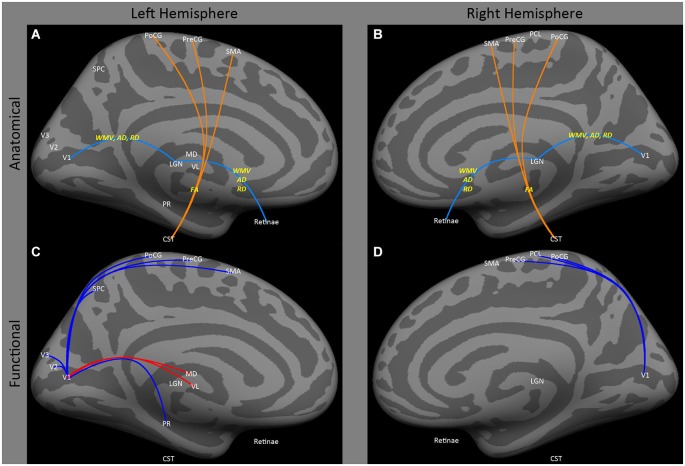
**Medial view of left and right hemispheres**. The upper panels **(A,B)** are a summary of major findings from the human anatomical connectivity literature, as described in the main text. Increases in anatomical connectivity as a result of early blindness or anophthalmia are shown in orange; decreases in connectivity are shown in teal; types of anatomical measurements are shown in yellow. The lower panels **(C,D)** are a summary of major findings from the human functional correlations literature. Increases in functional correlations as a result of early blindness or anophthalmia are shown in red; decreases in functional correlations are shown in blue. Some lines represent findings from multiple studies. Explanations of abbreviations can be found in Section Abbreviations.

Both human and animal models show that early blindness leads to atrophy of the pathways from the retina to early visual cortex. Several animal models have shown atrophy of the connections between the eye and V1 after early onset blindness (Kahn and Krubitzer, 2002; Karlen et al., 2006), for review see Movshon and Van Sluyters (1981). Similarly, a variety of studies have consistently found decreased white matter volume, decreased axial diffusivity and increased radial diffusivity in the optic nerve/tract (Figures 1A,B: retinae ↔ LGN) and optic radiations (Figures 1A,B: LGN ↔ V1) within both early blind (Noppeney et al., 2005; Shimony et al., 2006; Yu et al., 2007; Ptito et al., 2008; Shu et al., 2009b) and anophthalmic (Bridge et al., 2009) individuals. Yu et al. (2007) also found increased fractional anisotropy in the cortical spinal tract in early blind individuals (Figures 1A,B: CST ↔ PoCG, PreCG, SMA).

It is unknown whether early blindness also leads to novel connections to occipital cortex from auditory subcortical structures. In early blind animal models, novel weak connections to V1 from auditory, motor and somatosensory thalamic areas have been observed (Karlen et al., 2006; Karlen and Krubitzer, 2009). Although in theory it may be possible to isolate novel tracts from the medial geniculate tract to early visual areas in humans (Devlin et al., 2006), to date there is no anatomical evidence for novel subcortical input to early visual areas in early blind humans.

### Callosal connections

It is not clear whether blindness results in a large-scale loss of inter-hemispheric occipital connectivity, though animal models show clear disruption of callosal connections at a local level. In the macaque, prenatal enucleation results in a reduction in the size of V1, which is accompanied by a dramatic *increase* in V2 callosal connectivity (Dehay et al., 1989). In humans, there is mixed anatomical data on the effects of early blindness on inter-hemispheric connections. Shimony et al. (2006) found no difference in mean diffusivity between 5 early blind subjects (3 retinopathy of prematurity (ROP)) and 7 seven sighted controls, but did find a barely significant reduction in relative anisotropy within the ventral half of the splenium. Yu et al. (2007) found a significant reduction in fractional anisotropy within the splenium across 17 early blind subjects and their sighted controls. However, Bock et al. (2013), examining six early blind and six anophthalmic subjects, found no difference in fractional anisotropy in either the splenium or in the callosum (as a whole) compared to sighted controls, although a slight decrease in mean diffusivity was found in the splenium of anophthalmic subjects. Interestingly, Bridge et al. (2009), using tract-based spatial statistics, found a decrease in fractional anisotropy in *non*-visual anterior portions of the callosum in the same anophthalmic subjects.

Animal models clearly show that deprivation disrupts the local topography of callosal connections. Studies in V2 of early blind animals show increased “patchiness” and a spreading of the callosal connections between hemispheres into regions of cortex that are acallosal in sighted control animals (Olavarria et al., 1987; Dehay et al., 1989; Innocenti and Berbel, 1991; Olavarria and Van Sluyters, 1995; Olavarria and Hiroi, 2003; Innocenti and Price, 2005). However, this disruption seems to be local—often on the order of microns. Indeed, major topographical features, including the restriction of callosal fibers to the border between visual areas representing the vertical meridian, can still be recognized in animals lacking visual input (Bock et al., 2010, 2012; Bock and Olavarria, 2011). Consistent with this, gross topographical organization within the callosum, as measured using diffusion weighted imaging, does not seem to be disrupted in early blind or anophthalmic individuals (Bock et al., 2013).

Thus, overall the diffusion literature suggests neither the strength nor the macro-scale topographic organization of callosal connections are dramatically affected by early blindness.

### Intra-hemispheric cortico-cortical connectivity

The main changes in cortico-cortical white matter that have been observed as a result of early blindness using diffusion weighted imaging suggest attenuation of occipital to temporal connections, and an increase in the white matter volume of connections between occipital and frontal cortex.

**Figure 2:**
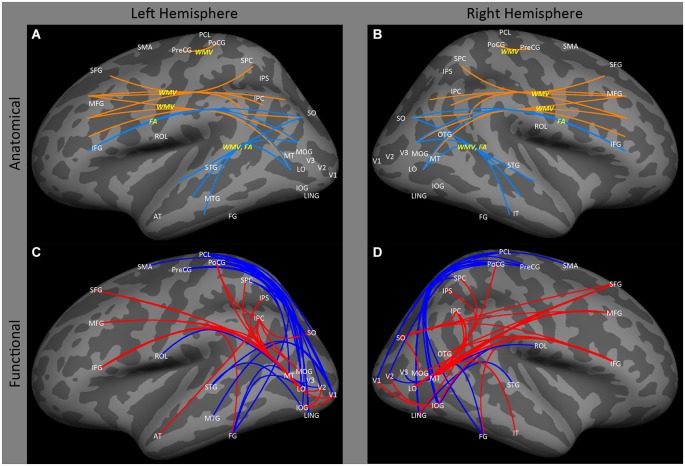
**Lateral view of left and right hemispheres**. The upper panels **(A,B)** are a summary of major findings from the human anatomical connectivity literature, as described in the main text. Increases in anatomical connectivity as a result of early blindness or anophthalmia are shown in orange; decreases in connectivity are shown in teal; types of anatomical measurements are shown in yellow. The lower panels **(C,D)** are a summary of major findings from the human functional correlations literature. Increases in functional correlations as a result of early blindness or anophthalmia are shown in red; decreases in functional correlations are shown in blue. Some lines represent findings from multiple studies. Explanations of abbreviations can be found in Section Abbreviations.

Ptito et al. (2008), using voxel based morphometry, found that early blindness resulted in reductions in white matter volume in tracts of the inferior longitudinal fasciculus connecting regions within lateral occipital and temporal cortex. Similarly, Shu et al. (2009b) found reductions in fractional anisotropy (identified on the basis of graph connectivity), in similar tracts (Figures 2A,B: SO, MOG and MT ↔ STG and MTG). As described below, this finding is consistent with data showing a reduction of functional correlations between occipital and temporal areas.

There is also some evidence for an increase in white matter volume within the tracts between lateral occipital and prefrontal cortex. Ptito et al. (2008) reported significant increases in white matter volume in both occipito-frontal (Figures 2A,B: SO, MOG and MT ↔ IFG) and superior longitudinal (Figures 2A,B: SPC, and IPC ↔ SFG) fasciculi. Once again, this is consistent with the functional correlations literature, which shows enhanced correlations between these areas. However, it is worth noting that Shu et al. (2009b) found a *reduction* in fractional anisotropy within the inferior occipito-frontal tract (Figures 2A,B: SO, MOG and MT ↔ IFG).

Finally, Noppeney et al. (2005) have reported increased volume in white matter tracts between motor and somatosensory cortex (Figures 2A,B: PoCG ↔ PreCG).

### Summary of anatomical findings

In summary, there is a consensus across multiple human and animal studies that early blindness results in atrophy of the pathways from the eye to V1. There is also evidence for changes in anatomical connectivity within a few major cortico-cortical tracts, particularly a weakening of connections between occipital and temporal cortex (indicated by increases in both white matter volume and fractional anisotropy), and an increase in the white matter volume of connections between occipital and frontal cortex.

The data for changes in cortico-cortical anatomical connectivity resulting from early blindness are perhaps less dramatic and extensive then might have been expected given that alterations in experience that are presumably far less dramatic, such as juggling (Scholz et al., 2009), video-game playing (e.g., Colom et al., 2012) and learning golf (Bezzola et al., 2011) induce measurable anatomical changes in gray and white matter. This may be because comparisons of white matter changes between blind and sighted individuals necessarily involve group comparisons, and variability in measurements such as fractional anisotropy is far greater when measured in a between-subject as compared to a within-subjects design (Veenith et al., 2013). Based on estimates of variability from Veenith et al., power analyses suggest that a 5% difference in fractional anisotropy (similar to that produced by juggling Scholz et al., 2009) within the posterior portion of the corpus callosum would only require 4 subjects/group to reliably detect in a within-subject design (.9 power) but would require 180 subjects/group to obtain similar power in a between-subject design. Cortico-cortical tracts also show significant between-subject variability, for example, within the inferior and superior longitudinal fasciculus, a 5% difference in fractional anisotropy (.9 power) would require a sample size of 3–4 subjects/group in a within-subject design as compared to 12–19 subjects/group in a between-subject design. Because of the difficulty of recruiting blind subjects with relatively homogenous visual histories, many of the studies cited above used moderate numbers of subjects (11 blind and 42 sighted, Noppeney et al., 2005; 11 blind and 21 sighted, Ptito et al., 2008; 17/group, Shu et al., 2009a). Thus, it seems possible that larger sample sizes (perhaps through a multi-center study) might better reveal anatomical differences in connectivity as a result of early blindness.

## Functional correlations

Functional correlation (or “connectivity”) analyses rest on the notion that correlated activity between different brain regions reflect functionally important correlations in neuronal firing (for a review see Fox and Raichle, 2007). This assumption rests on observations that functional correlations reveal a canonical organization of brain network architecture that reflects direct and indirect connectivity (Biswal et al., 1995; for review see Damoiseaux and Greicius, 2009; e.g., Honey et al., 2009; Smith et al., 2009; Yeo et al., 2011; Marcus et al., 2013; Goñi et al., 2014). However, systemic low frequency oscillations of non-neural origin also contribute significantly to resting state signals, such that even the most accepted resting state networks are likely to contain significant peripheral physiological contributions that include respiration and cardiac pulsation as well as signals that remain of unknown origin (Birn et al., 2008; Honey et al., 2009; Tong and Frederick, 2014). This seems to be a particular concern for primary sensorimotor, auditory and visual networks (Tong et al., 2013), due to their high blood capillary density and/or vascular density (Harrison et al., 2002).

It is also worth noting that, in the case of blindness, changes in functional correlations (described below) need not reflect changes in connectivity *per se*, but rather within-region changes in functional response profiles (Hermundstad et al., 2013). As a theoretical example, given that both hMT+ and auditory cortex respond to auditory stimulation in blind individuals (Watkins et al., 2013), their responses, especially in a noisy scanner, might be expected to be more strongly correlated simply as a consequence of having more similar stimulus preferences.

### Subcortical

Despite reduced anatomical connectivity in the optic nerve/tract and optic radiations having been replicated in several studies (as described above), no evidence has been found for reductions in functional correlations between “visual” cortical and subcortical areas. Bedny et al. (2011) actually found an *increase* in functional correlations between lateral and medial occipital regions and ventral lateral and medial dorsal thalamic nuclei in the left hemisphere (Figures 1C,D: MD and VL ↔ CAL), though these data were based on “rest” periods within a task paradigm, so may have been driven by task-related effects.

### Occipital cortex

The effects of blindness on ipsilateral functional correlations between visual areas are not particularly clear. Burton et al. (2014) found that blindness had relatively little effect on ipsilateral occipital cortex correlations, especially when compared to the effect on inter-hemispheric correlations. Bedny et al. (2010) found a *decrease* in ipsilateral correlations between MT and V2–3, between V1 and V2–3, and between lateral occipital cortex (LO and MT) and inferior temporal cortex (Figures 2A,B: MT and LO ↔ FG) in the left hemisphere. However, Qin et al. (2013) reported an *increase* in functional correlations between early (V1 and V2) and lateral occipital higher level visual areas such as the inferior occipital gyrus and the lateral occipital cortex (Figures 2A,B: V1 and V2 ↔ IOG and LO).

**Figure 3:**
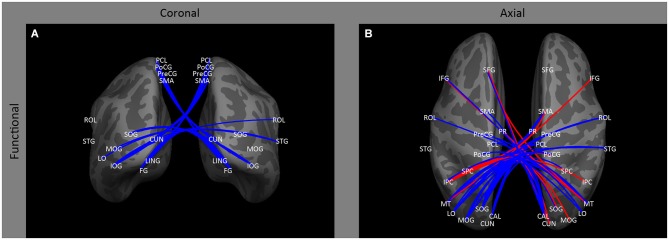
**Inter-hemispheric functional correlations.** Coronal **(A)** and axial **(B)** views of inter-hemispheric functional correlations. Increases in functional correlations as a result of early blindness or anophthalmia are shown in red, decreases in functional correlations are shown in blue. Some lines represent findings from multiple studies.

In contrast to the relatively small effect on ipsilateral occipital connectivity, a variety of studies have shown that early blindness (Bedny et al., 2011; Qin et al., 2013; Burton et al., 2014) and anophthalmia (Watkins et al., 2012) results in a *decrease* in inter-hemispheric functional correlations for resting state signals within occipital cortex between both homologous and non-homologous areas, with the difference between subject groups tending to increase across the visual hierarchy (Figure 3B: CAL ↔ CAL, LO, MOG, MT, SOG; LO ↔ LO; MT ↔ MT; SOG ↔ SOG, IOG). This decrease in correlations across entire areas may be accompanied by some topographical disruption: Butt et al. (2013) found a small weakening of the topographic organization of V1 inter-hemispheric correlations in early blind individuals.

Inter-hemispheric resting state correlations are difficult to interpret even in sighted individuals, making the reduction in these correlations in early blind individuals still more mysterious. There are three reasons to believe that these correlations are not primarily driven by callosal fibers representing the midline, even in sighted individuals. First, the strength of the correlations are not stronger for cortical regions representing the midline (Butt et al., 2013). Second, between-hemisphere resting correlations are preserved in subjects with chronic callosal disconnection (Corsi-Cabrera et al., 2006; Uddin et al., 2008; Tyszka et al., 2011), and finally correlations are strongest between regions of cortex representing *symmetrical* rather than corresponding locations in visual space.

One possibility is that these correlations are driven by subcortical connections via the superior colliculus (Savazzi et al., 2007). If so, these reductions in functional correlations are consistent with the known atrophy of early visual pathways observed in early blind individuals, as described above. However, according to this explanation, one would expect the reduction in correlations found in blind individuals to be greater in V1 than in higher visual areas, whereas the opposite pattern of results has been observed, with differences between early blind or anophthalmic and sighted subjects increasing along the visual hierarchy (Bedny et al., 2011; Watkins et al., 2012; Qin et al., 2013; Burton et al., 2014).

A second interpretation rests on the observation that resting-state correlations are likely a combination of two separate components whose relative strengths vary across the visual hierarchy. The first component is non-neural low frequency oscillations, originating from vasculature that symmetrically stems from the posterior cerebral artery (Tong et al., 2013; Tong and Frederick, 2014). These non-neural resting-state correlations are likely to be unaffected, or potentially even strengthened, by blindness (Wanet-Defalque et al., 1988; Veraart et al., 1990; Uhl et al., 1993; De Volder et al., 1997; Weaver et al., 2013). In V1, which is extremely highly vascularized, these non-neural components are likely to dominate the resting state signal, leading to little or no difference in resting-state correlations between the blind and sighted. In contrast, higher level areas are less heavily vascularized, and non-neural components contribute less to the resting-state signal (Harrison et al., 2002; Tong et al., 2013; Tong and Frederick, 2014). If the neural component in the inter-hemispheric resting-state signal increases in strength across the visual hierarchy (as would be expected from receptive field organization) and is attenuated by blindness then this explanation would predict three somewhat counterintuitive findings in the literature. First, the non-neural component based on a symmetric vasculature pattern predicts the observation that resting-state correlations are strongest between regions of visual cortex that represent symmetrical rather than corresponding locations in visual space in both early blind and sighted individuals. Second, the decline in the contribution of the non-neural component across the visual hierarchy predicts the somewhat counterintuitive finding that resting state correlations decrease (Watkins et al., 2012) or are non-monotonic (Burton et al., 2014) across the visual hierarchy in sighted subjects, even as the proportion of receptive fields receiving information from both left and right V1 increases. Finally, this explanation would predict why there is little difference between blind and sighted inter-hemispheric functional correlations in V1, but blind and anophthalmic subjects show increasingly attenuated inter-hemispheric functional correlations for higher visual areas (Bedny et al., 2011; Watkins et al., 2012; Qin et al., 2013; Burton et al., 2014).

Finally, regardless of whether inter-hemispheric correlations in visual areas are driven by subcortical input or a combination of non-neural and cortical neural components, it is possible that the reduction in inter-hemispheric correlations noted in blind subjects might be influenced by lateralized enhanced input from other regions of cortex. Some studies have found that left-lateralized language areas in the frontal and parietal lobes display increased functional correlations with left occipital cortex in blind (Butt et al., 2013) and anophthalmic subjects (Watkins et al., 2012), though this has not been replicated by Burton et al. (2014).

### Occipital to temporal cortex

Consistent with the loss of anatomical connectivity described above, several studies have observed *decreased* functional correlations between occipital cortex and ipsilateral and contralateral temporal regions. A variety of studies (Liu et al., 2007; Bedny et al., 2010, 2011; Butt et al., 2013; Burton et al., 2014) have shown *decreased* functional correlations between early visual areas and ipsilateral regions of occipital temporal and temporal cortex (Figures 2C,D: V1, V4, MT↔43, STG and MTG) associated with auditory and language processing (Bedny et al., 2011; Watkins et al., 2012) in early blind and anophthalmic individuals. Similarly, Burton et al. (2014; also see Yu et al., 2008) found *decreased* functional correlations between contralateral visual and auditory cortices (Figures 3A,B: V1↔STG). However, *increased* functional correlations have been noted between LO and IT in the right hemisphere in anophthalmic individuals (Watkins et al., 2012; Figures 2A,B: LO ↔ IT).

### Occipital to somatosensory cortex

Early blindness results in reduced functional correlations between occipital and somatosensory cortex. Several studies (Liu et al., 2007; Yu et al., 2008; Bedny et al., 2010, 2011; Qin and Yu, 2013; Burton et al., 2014) have found a *decrease* in functional correlations between a wide range of occipital and ipsilateral sensorimotor regions, especially primary somatosensory and primary motor areas (Figures 1C,D: V1 ↔ PoCG, PCL, PreCG; Figures 2C,D: V1 to VP, MOG, IOG, LO, LING, SOG and FG ↔ PCL, PoCG, PreCG and SMA). Only one study (Sani et al., 2010) found the opposite—*increased* functional correlations between hMT+ and somatosensory areas (Figures 2C,D: MT ↔ PoCG and SPC).

Similarly, several studies (Liu et al., 2007; Bedny et al., 2011; Qin et al., 2013; Burton et al., 2014) have found evidence of *decreased* functional correlations between occipital cortex and contralateral multisensory, and sensorimotor areas (Figure 3A: IOG, SOG, MOG, LING↔STG; MOG and IOG ↔ ROL; IPC ↔ STG; V1 to LO ↔ S1; MOG ↔ STG and ROL).

### Parietal cortex

Early blindness results in increased correlations between occipital and parietal cortex. Several studies (Sani et al., 2010; Collignon et al., 2011; Leo et al., 2012; Watkins et al., 2012) find *increased* correlations from occipital to ipsilateral parietal cortex in early blind and anophthalmic subjects (Figures 2C,D: MT, LO, SO, MOG, LING, AT, PoCG, SO; SO ↔ IPC, SFG and MOG↔ IPC, IPS and SPC).

Similarly, correlations between visual areas and contralateral parietal occipital cortex (Collignon et al., 2011; Leo et al., 2012; Burton et al., 2014) tend to be *increased* (Figures 3A,B: CUN ↔ IPC, SFG; IPC ↔ SOG, MOG, PoCG and IPC) by early blindness.

However, in early blind individuals (Bedny et al., 2011; Leo et al., 2012; Qin et al., 2013; Burton et al., 2014) there is evidence of *decreased* correlations between homologous parietal visual areas (Figures 3A,B: right VP ↔ left VP, right IPC ↔ left IPC), and between contralateral parietal and occipital cortex (Figures 3A,B: IPC ↔ CAL, LO, PoCG, STG; SPC ↔ MT; PoCG ↔ CAL, LO, MOG, MT, SOG). This finding is somewhat analogous to the finding of reduced correlations between homologous and non-homologous visual areas in early blind individuals, described above.

### Pre-frontal and frontal cortices

Finally, a wide variety of studies have found *increased* functional correlations between visual areas (especially higher order areas) and ipsilateral and contralateral prefrontal (Sani et al., 2010; Bedny et al., 2011; Collignon et al., 2011; Watkins et al., 2012; Burton et al., 2014; Renier et al., 2014; Figures 2C,D: MT, FG, MOG, OTG and LO-IFG, 44–45, 47) and frontal (Liu et al., 2007; Bedny et al., 2010, 2011; Sani et al., 2010; Collignon et al., 2011; Watkins et al., 2012; Burton et al., 2014) cortex (Figures 2C,D: FG, IOG, SOG, LO, MT and OTG-46, 6, 8, 9, IFG, MFG, SFG).

## Discussion

In summary, the most well-established changes in anatomical connectivity as a result of blindness include atrophy of the pathways from the retina to early visual cortex, attenuation of occipital to temporal connections, and strengthening of connections between occipital and frontal cortex.

The main effect of early blindness on functional correlations seem to be reduced inter-hemispheric functional correlations within occipital and parietal regions, *decreased* functional correlations between occipital and ipsilateral and contralateral sensorimotor cortex, *decreased* functional correlations between visual areas and ipsilateral and contralateral temporal regions associated with auditory processing (consistent with the anatomical data), and *increased* functional correlations between early visual areas and areas associated with higher level cognitive functions including parietal, prefrontal and frontal cortex, (again consistent with the anatomical data).

It has been suggested that these findings are evidence for recruitment of occipital cortex for higher level cognitive functions, such as memory, attention and cognitive control (Burton et al., 2014), and/or language (Bedny et al., 2011; Watkins et al., 2013), rather than simple sensory functions. Certainly, cross-modal responses in occipital cortex in blind individuals tend to be larger when attention is engaged (Stevens et al., 2007; Weaver and Stevens, 2007) or the task contains a working memory component (Burton et al., 2010; see Burton et al. (2014), for a review). However this is generally true of sensory cortex, and robust auditory and tactile responses have been observed in occipital cortex as a result of early blindness which cannot easily be explained as representing attentional, cognitive control or working memory operations (Mahon et al., 2009; Collignon et al., 2011; Huber et al., 2014; Jiang et al., 2014).

Moreover, if the enhanced functional correlations between parietal/frontal cortices and occipital cortex represents recruitment of occipital cortex for cognitive control operations, one would expect that the regions of occipital and frontal cortex that share enhanced functional correlations would have similar functional roles. This was not observed in a recent study (Bedny et al., 2013) explicitly comparing functional responses to an n-back working memory task and a language task. A variety of occipital regions showed increased functional correlations with regions of frontal cortex, mainly to prefrontal regions that responded to working memory load. However, many of these occipital regions did not themselves show functional responses to working memory load, as would be expected if they had been recruited for that purpose. Furthermore, both the Bedny et al. (2013) study and that of Burton et al. (2014) show an unselective pattern of connectivity between prefrontal and occipital areas, whereby wide regions of prefrontal cortex were connected to most of occipital cortex. If occipital cortex was being recruited for cognitive control operations, one might expect a more selective pattern of connectivity that respected the different functional roles of prefrontal cortex.

An alternative possibility is that the *increase* in connectivity between occipital cortex and regions associated with cognitive control reflects increased gating of occipital responses by frontal cortex. Both this phenomenon, and the somewhat counterintuitive finding of a *decrease* in functional correlations between occipital cortex and other sensory areas, can be predicted by a model that explains cross-modal plasticity as a result of blindness in terms of a ME architecture, as described below.

### The mixture-of-experts architecture

**Figure 4:**
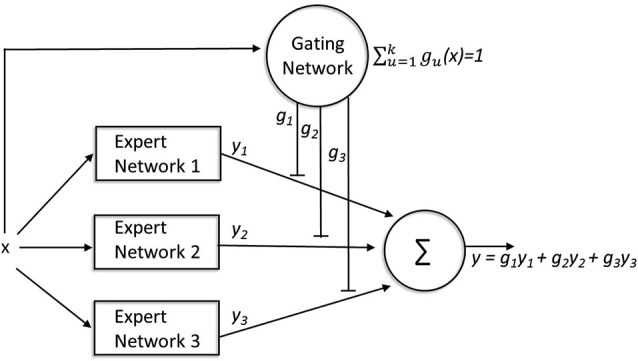
**A mixture-of-experts architecture**. The expert networks compete to learn tasks while the gating network mediates the competition. For every input (x), the gating network receives information about the performance of all of the expert networks (y_1,2,3_) involved in solving the task, and each expert network”s output is compared with the target output (y). The weights gating the output of each expert network (g_1,2,3_) are modified based on the relative performance of that expert network (compared to the other experts) for that input pattern. These gating weights not only determine the extent to which the output of each network contributes to the final output, but also modulate learning within each network such that more learning occurs within those expert networks that contribute more heavily to the final output.

The ME modular architecture describes how a number of “experts” (or modules) compete to learn different tasks. For the purposes of illustration, we describe the classic mixture-of-experts (ME) model described by Jacobs et al. (1991), see Figure 4. This instantiation should simply be considered as an example: many other architectures make similar predictions (Yuksel et al. (2012) for a review). The traditional ME architecture consists of two types of networks: expert networks and a gating network. Expert networks compete to learn tasks, while the gating network mediates the competition. The final output of the full network is a weighted average (with gating weights g_1_, g_2_, g_3,_ … g_n_) of the outputs (y_1_, y_2_, y_3,_ … y_n_) of each expert network. For each input, x, the gating network receives information about the performance of all of the expert networks in solving the task (finding the correct y for that x) and each expert network”s output is compared with the target output. Learning occurs in two ways. First, the weights gating the output of each expert network are modified based on the relative performance of that expert network (compared to the other experts) for that input pattern. This is implemented by forcing the activation of the output units to be nonnegative and sum to 1. Thus, the gating weights determine the extent to which the output of each network contributes to the final output, so that on future trials with similar input the most accurate expert will have a larger influence on the final response than less accurate experts. Learning also occurs *within* each network: this learning is also modulated by the gating weights such that more learning occurs within those expert networks that contribute more heavily to the final output.

One critical difference between traditional Hebbian competitive learning models and the ME architecture is that, in the former, the units of a single network compete for the right to respond maximally to a given subset of input patterns, thereby learning to partition the *input* space. In contrast, the ME model clusters *input-output* patterns or “tasks” into natural groupings. The architecture imposes modularity, such that each expert learns a different set of tasks. In fact, in this network architecture, the *goal of the gating network is to guide the expert networks towards dividing the task-space in such a way as to minimize correlation between expert networks*. One advantage of this is that weight changes within each expert network during the process of learning are localized to the expert networks that perform well on that task; this decoupling of the weight changes across different expert networks reduces interference between tasks.

### ME predicts task-specific plasticity

The emphasis of the ME architecture on dividing the “task” rather than the “input” space is consistent with the observation of Burton et al. (2014) that while a wide variety of tasks elicit cross-modal responses, the perceptual advantages and cross-modal responses observed within early blind individuals seem to be highly task-specific (Sadato et al., 1996; Burton et al., 2002; Gizewski et al., 2003), rather than simply representing generically enhanced sensory abilities.

### ME predicts “metamodal” reorganization

One attractive aspect of the ME framework is that it provides a model for one of the most influential theories about the organization of cross-modal plasticity as a result of blindness: the “functional constancy” or “metamodal” (Pascual-Leone and Hamilton, 2001; Pascual-Leone et al., 2005) hypothesis that cortical areas maintain their computational specialization following the loss of a sense, but shift their preferred input modality. According to this hypothesis, brain areas are specialized for a certain type of “information processing” or “task” (e.g., hMT+ computes the motion of objects), and the relative weights of different input modalities (e.g., vision/touch/audition) within that area are related to how useful information in that modality is for that particular task. According to the metamodal hypothesis, hMT+ is primarily driven by vision in sighted individuals simply because vision is a very reliable source of motion information. In the absence of vision, hMT+ will continue to be specialized for processing motion information, but the weights of non-visual modalities that provide motion information will be increased. There are now a number of studies providing evidence for “functional constancy” (a change in modality without a change in functional role) in blind individuals. For example, hMT+ responds to auditory and tactile motion (e.g., Saenz et al., 2008; Bedny et al., 2010; Wolbers et al., 2011), object-sounds are represented in brain regions associated with visual object recognition (Amedi et al., 2007; Mahon et al., 2009), reading Braille elicits brain responses in the visual word-form region (Striem-Amit et al., 2012), and a variety of visual areas such as the fusiform face area (Gougoux et al., 2009), tool area (Mahon et al., 2009) and extrastriate body area (Striem-Amit and Amedi, 2014) retain similar functional selectivity preferences even in early blind individuals. It remains to be seen whether the improved behavioral performance found in early blind individuals for other specific auditory (Lessard et al., 1998; Röder et al., 1999; Gougoux et al., 2005) and tactile (Van Boven et al., 2000; Goldreich and Kanics, 2003; Alary et al., 2009) tasks is due to the recruitment of regions of visual cortex specialized for analogous visual tasks.

Finally, one of the proposed motivations for “functional constancy” is that it allows for computational tasks to remain within brain regions whose innate characteristics (whether it be fine scale neuronal structure or the pattern of connections to other cortical areas) are well suited to that task, regardless of the change in input modality (Pascual-Leone and Hamilton, 2001; Pascual-Leone et al., 2005; Mahon et al., 2009). Interestingly, the mixture-of-experts model has specifically been demonstrated to have the capability to assign tasks to cortical regions on the basis of differences in innate architecture within individual experts (Jacobs and Kosslyn, 1994; Jacobs, 1997). Thus, the “ME” framework, because of its emphasis on *task* rather than input, provides a very natural computational implementation for metamodal reorganization.

### ME predicts loss of functionality in non-deprived areas

It is sometimes assumed in the literature that the role of cross-modal responses in occipital cortex is to *augment* responses in non-deprived auditory and somatosensory cortices. However, another possibility is that occipital cortex competes with non-deprived regions of cortex to represent tasks. In support of this possibility, two studies have shown that cross-modal plasticity in specific tasks is associated with a loss of functionality in non-deprived areas in early blind subjects. The recruitment of pericalcarine cortex by Braille reading in early blind individuals may be accompanied by weaker responses in somatosensory areas for Braille reading (Sadato et al., 1998), even though blind subjects show equal or enhanced responses in somatosensory areas compared to sighted individuals for other types of tactile task (Burton et al., 2004, 2010). Analogously, the recruitment of visual area hMT+ for auditory motion processing in early blind individuals (Saenz et al., 2008) is accompanied by a loss of selectivity to auditory direction of motion stimuli in regions of auditory cortex (e.g., planum temporale) that are motion selective in sighted subjects (Jiang et al., 2014). Thus, for these particular tasks, the recruitment of occipital cortex seems to supplant, rather than augment, processing in non-deprived areas. Because the ME architecture enforces competition between modules to represent tasks, it predicts this very outcome.

### ME predicts lowered functional correlations between occipital and non-deprived sensory cortices

As described above, it is somewhat counterintuitive that occipital areas show *reduced* anatomical connectivity (indicated by reduced fractional anisotropy and white matter volume within the inferior fasciculus) and reduced functional correlations with both sensorimotor and temporal cortices given that occipital cortex shows widespread responses to auditory and tactile stimuli in early blind individuals (e.g., Lewis et al., 2010 for reviews see Kupers and Ptito, 2014; Renier et al., 2014).

One response to this apparent contradiction has been to propose that occipital cortex may be heavily modulated by attention and/or working memory load (Burton et al., 2014). But even if sensory responses were more heavily modulated by attention, the increased similarity of input stimulus preferences between occipital and sensorimotor and auditory cortices would predict *higher* rather than lower correlations between these regions (Hermundstad et al., 2013), while the opposite is observed in early blind individuals.

Interestingly, the ME architecture can explain a *decrease* in functional correlations despite increased overlap in stimulus input preferences. Take the finding of reduced functional correlations between sensorimotor and visual cortex in blind individuals. In sighted subjects, the low level input to sensorimotor and visual cortex is likely to be relatively de-correlated (there is likely to be almost no correlation between individual V1 and S1 neurons), and the early computations involved in basic sensorimotor and visual tasks (e.g., cup grasping vs. recognizing a cup) are similarly likely to be orthogonal to each other. Indeed, in sighted subjects, primary visual, auditory and sensory areas only show weak functional correlations with each other (Burton et al., 2014). Only at higher (object-level) stages of processing are these two very different computations likely to be multi-modally integrated, in a highly task-dependent manner. In contrast, within early blind subjects functional activation of these areas suggests that tactile tasks are assigned to both somatosensory *and* occipital cortex. According to an ME architecture, these areas then compete to represent particular tasks. As one area (e.g., occipital cortex) becomes an expert for a given task (perhaps Braille reading), there will be corresponding changes in other areas (e.g., somatosensory cortex) that shift their representations *away* from that task and towards other tactile tasks, thereby *maximally de-correlating the outputs of the two “expert networks”*. Thus, the ME architecture naturally predicts lower functional correlations and possibly reduced anatomical connectivity between occipital and somatosensory/auditory cortex in blind individuals, despite more highly correlated sensory *input* into the two areas.

### ME predicts enhanced connectivity between occipital and pre-frontal/frontal areas

Finally, as described above, a wide variety of studies show increased anatomical (indicated by increased white matter volume) connectivity and higher functional correlations between occipital and frontal regions. One possibility, as described above, is that these increased functional correlations represent the recruitment of visual cortex for cognitive control/language functions, similar to those performed by frontal cortex (Bedny et al., 2011, 2012; Watkins et al., 2013; Burton et al., 2014). In contrast, according to the ME architecture, increased functional correlations and enhanced anatomical connectivity between frontal and occipital regions actually represent increased gating demands rather than a shared functional role. It seems plausible that the contextual demands required in gating information in a task specific manner (Frank and Badre, 2012) are more complex in early blind than in sighted individuals. Consistent with this hypothesis, auditory and somatosensory regions also tend to show increased functional correlations with regions associated with cognitive control operations as a result of early blindness, though these increased functional correlations failed to reach significance within most individual ROIs (Burton et al., 2014).

## Conclusions

As described above, there is a growing amount of evidence about the neuroanatomical and functional changes that result as a consequence of blindness. However, our understanding of these findings has tended towards the descriptive. Here we propose the ME framework as an example of a more mechanistic model, and show how this model can reconcile a wide range of functional and anatomical data.

## Conflict of interest statement

The authors declare that the research was conducted in the absence of any commercial or financial relationships that could be construed as a potential conflict of interest.

## Footnotes

^1^as well as soothsaying Apollodorus (C2nd A.D.). in: Apollodorus. The Library. Books 1-3.9.).

## Abbreviations

AD: axial diffusivity
FA: fractional anisotropy
RD: radial diffusivity
WMV: white matter volume
AT: anterior temporal cortex
CST: corticospinal tract
FG: fusiform gyrus
IFG: inferior frontal gyrus
IOG: inferior occipital gyrus
IPC: intraparietal cortex
IPS: intraparietal sulcus
LGN: lateral geniculate nucleus
LING: lingual gyrus
LO: lateral occipital area
MD: medial dorsal nucleus
MFG: middle frontral gyrus
MOG: middle occipital gyrus
MT: middle temporal area
MTG: middle temporal gyrus
PCL: paracentral lobule
PreCG: precentral gyrus
PoCG: postcentral gyrus
PR: perirhinal cortex
ROL: rolandic cortex
SFG: superior frontal gyrus
SMA: supplementary motor area
SO: superior occipital cortex
SPC: superior parietal cortex
STG: superior temporal gyrus
V1: Brodmann area 17
V2: Brodmann area 18
V3: Brodmann area 19
VL: ventral lateral nucleus.
